# The Transcriptomic Profile of Monocytes from Patients With Sjögren’s Syndrome Is Associated With Inflammatory Parameters and Is Mimicked by Circulating Mediators

**DOI:** 10.3389/fimmu.2021.701656

**Published:** 2021-08-03

**Authors:** Ana P. Lopes, Cornelis P. J. Bekker, Maarten R. Hillen, Sofie L. M. Blokland, Anneline C. Hinrichs, Aridaman Pandit, Aike A. Kruize, Timothy R. D. J. Radstake, Joel A. G. van Roon

**Affiliations:** ^1^Center for Translational Immunology, University Medical Center Utrecht, Utrecht University, Utrecht, Netherlands; ^2^Department of Rheumatology and Clinical Immunology, University Medical Center Utrecht, Utrecht University, Utrecht, Netherlands

**Keywords:** Sjögren’s syndrome, monocytes, transcriptome, WGCNA, type-I interferons, systemic mediators

## Abstract

Primary Sjögren’s syndrome (pSS) is a systemic autoimmune disease characterized by infiltration of the exocrine glands and prominent B cell hyperactivity. Considering the key role of monocytes in promoting B cell hyperactivity, we performed RNA-sequencing analysis of CD14^+^ monocytes from patients with pSS, non-Sjögren’s sicca (nSS), and healthy controls (HC). We demonstrated that the transcriptomic profile of pSS patients is enriched in intermediate and non-classical monocyte profiles, and confirmed the increased frequency of non-classical monocytes in pSS patients by flow-cytometry analysis. Weighted gene co-expression network analysis identified four molecular signatures in monocytes from pSS patients, functionally annotated for processes related with translation, IFN-signaling, and toll-like receptor signaling. Systemic and local inflammatory features significantly correlated with the expression of these signatures. Furthermore, genes highly associated with clinical features in pSS were identified as hub-genes for each signature. Unsupervised hierarchical cluster analysis of the hub-genes identified four clusters of nSS and pSS patients, each with distinct inflammatory and transcriptomic profiles. One cluster showed a significantly higher percentage of pSS patients with higher prevalence of anti-SSA autoantibodies, interferon-score, and erythrocyte sedimentation rate compared to the other clusters. Finally, we showed that the identified transcriptomic differences in pSS monocytes were induced in monocytes of healthy controls by exposure to serum of pSS patients. Representative hub-genes of all four signatures were partially inhibited by interferon-α/β receptor blockade, indicating that the circulating inflammatory mediators, including type I interferons have a significant contribution to the altered transcriptional profile of pSS-monocytes. Our study suggests that targeting key circulating inflammatory mediators, such as type I interferons, could offer new insights into the important pathways and mechanisms driving pSS, and holds promise for halting immunopathology in Sjögren’s Syndrome.

## Introduction

Primary Sjögren’s syndrome (pSS) is a systemic autoimmune disease characterized by lymphocytic infiltration of the salivary and lachrymal glands leading to glandular destruction and dysfunction and B cell hyperactivity (1). Although pSS pathogenesis remains to be fully uncovered, the important contribution of monocytes is evident, not only in the initial immune response but also in the maintenance of chronic inflammation (2–4). Monocytes are highly specialized in phagocytosis and antigen presentation, secrete a large range of different cytokines and chemokines, and migrate to the tissues in response to infection and injury. Once recruited to tissues, monocytes are capable of differentiating into macrophages and dendritic cells and thus contribute to local inflammation (5, 6).

Circulating blood monocytes are a heterogeneous population with a key role in regulation of inflammation (7), which can be subdivided into three major subsets: the classical monocytes (CD14^+^CD16^-^), the intermediate monocytes (CD14^+^CD16^+^), and the non-classical monocytes (CD14^-^CD16^+^), with the majority of the intermediate and non-classical monocytes emerging sequentially from the pool of classical monocytes (8, 9). Classical monocytes are primed for phagocytosis, innate sensing/immune responses, and migration, and are known to be important scavenger cells (10, 11). Intermediate monocytes represent a transitional population between the classical and non-classical monocyte subsets, with the highest capacity to present antigen (10), and induce CD4^+^ T cell proliferation (12). Non-classical monocytes have a pro-inflammatory behavior, secrete inflammatory cytokines in response to infection, and are involved in antigen presentation and T cell stimulation (10, 13). Recent studies have shown that this subset also contributes to the pathogenesis of several diseases, including autoimmune diseases like systemic lupus erythematosus (SLE) (14, 15). This contribution seems to be related with their patrolling behavior, Fc receptor-mediated phagocytosis, and higher secretion of pro-inflammatory cytokines, including tumor necrosis factor-alpha (TNF-α) and interleukin (IL)-1β (10, 16).

Several pieces of evidence point towards the contribution of monocytes to the immunopathology of Sjögren’s syndrome. The frequency of intermediate and non-classical monocytes (3, 17) is increased in the circulation of pSS patients, pointing to their activation and differentiation. In-line with this, the intermediate monocyte subset is increased in pSS salivary glands and their numbers directly correlated with the expression of IL-34 (17), a cytokine that acts as regulator of monocyte differentiation, proliferation, and survival (18). Further corroborating this, macrophages also accumulate in the salivary glands of pSS patients, and their presence was associated with lymphocytic infiltration and lymphoma (19). Furthermore, an abnormal activation of the NF-kB signaling pathway was observed in pSS monocytes (20), and the levels of pro-inflammatory mediators that are produced by monocytes and drive B cell activation, such as IL-6, type I interferon (IFN), B cell activating factor (BAFF), and chemokine C-X-C motif ligand 13 (CXCL13), are significantly increased in the inflamed tissues and circulation of pSS patients (21–24). Moreover, there is a marked upregulation of type I IFN-inducible genes (25) in pSS monocytes, reflecting the presence of a type I IFN signature in the salivary glands and circulation of pSS patients (26, 27). Type-I IFNs stimulate monocytes to produce inflammatory mediators (28) that drive B cell survival and maturation and ultimately sustains auto-antibody production (29). Thus, pSS monocytes seem to be activated, driving B cell hyperactivity, local inflammation, and ultimately immunopathology of pSS. Yet, the molecular mechanisms underlying the contribution of monocytes to pSS immunopathology have been poorly studied.

In-depth characterization of the dysregulated transcriptional profile of monocytes from pSS patients could offer new insights into the important pathways and mechanisms driving pSS, and ultimately might lead to the development of new therapeutic opportunities. We here exploited RNA-sequencing to investigate the transcriptional profile of circulating monocytes of pSS patients to unravel their role in the pathogenesis of pSS, and compared them to profiles of patients with non-Sjögren’s sicca (nSS) and healthy controls (HC).

## Materials and Methods

### Patients and Controls

Patients and controls were age and gender-matched and randomly allocated across the different experiments. All pSS patients fulfilled the AECG classification-criteria for pSS (30). Patients that did not fulfill the pSS classification-criteria, but presented with dryness-complaints without a known cause, in the absence of any generalized autoimmune disease were classified as non-Sjögren’s sicca (nSS) patients (**Table 1**). The study was approved by the medical ethics committee of the University Medical Center Utrecht (METC no. 13-697). All patients gave their written informed consent in accordance with the declaration of Helsinki.

**Table 1:** Characteristics of the patients and controls enrolled in the study.

	RNA sequencing			Whole blood		Serum stimulation	
	HC	nSS	pSS	HC	pSS	HC	pSS
N (M/F)	11 [1/10]	8 [0/8]	12 [2/10]	15 [0/15]	15 [0/15]	12 [0/12]	11 [0/11]
Age (yr.)	51 [29-59]	46 [24-69]	55 [26-76]	57 [25-63]	59 [22-81]	59 [52-71]	67 [33-75]
LFS (foci/4 mm^2^)	–	0.1 [0.0-0.6]	2.1 [1.0-4.0]	–	3.3 [1.0-6.4]	–	3.0 [1.0-6.5]
ESSDAI	–	–	5.0 [1.0-13]	–	5.0 [0.0-13]	–	9.0 [0.0-16]
ESSPRI	–	–	5.0 [1.0-8.0]	–	6.5 [2.0-9.0]	–	7.0 [3.0-9.0]
Schirmer (mm/5 min)	–	8.5 [1.5-32]	14 [0.0-28]	–	4.3 [0.0-24]	–	0.0 [0.0-15]
ANA (no. positive [%])	–	4 [50%]	10 [83%]	–	14 [93%]	–	11 [100%]
SSA (no. positive [%])	–	3 [38%]	9 [75%]	–	13 [87%]	–	11 [100%]
SSB (no. positive [%])	–	0 [0%]	4 [33%]	–	11 [73%]	–	11 [100%]
RF (no. positive [%])	–	1 [17%]	6 [60%]	–	7 [78%]	–	9 [90%]
Serum IgG (g/L)	–	13 [6.9-15]	16 [8.5-42]	–	12 [7.9-22]	–	19 [15-41]
ESR (mm/hour)	–	8 [5.0-23]	15 [3.0-77]	–	14 [2.0-27]	–	39 [11-76]
CRP (mg/L)	–	1.6 [0.2-5.2]	1.2 [0.0-4.2]	–	1.9 [0.6-22]	–	2.6 [0.9-8.1]
C3 (g/L)	–	1.2 [0.9-1.4]	1.0 [0.5-1.6]	–	1.0 [0.9-1.1]	–	1.0 [0.8-1.4]
C4 (g/L)	–	0.3 [0.2-0.4]	0.2 [0.1-0.4]	–	0.2 [0.1-0.3]	–	0.2 [0.0-0.3]
Not treated (no. [%])	–	7 [88%]	7 [58%]	–	7 [47%]	–	11 [100%]
Only HCQ (no. [%])	–	–	–	–	5 [33%]	–	–
Other (no. [%])	–	–	4 [33%]	–	3 [20%]	–	–

HC, healthy control; nSS, non-Sjögren’s sicca; pSS, primary Sjögren’s syndrome; LFS, lymphocyte focus score; ESSDAI, EULAR Sjögren’s syndrome disease activity index; ESSPRI, EULAR Sjögren’s syndrome patient reported index; ANA, anti-nuclear antibodies; SSA, anti-SSA/Ro; SSB, anti-SSB/La; RF, rheumatoid factor; ESR, erythrocyte sedimentation rate; CRP, C-reactive protein; HCQ, hydroxychloroquine. Other treatment group includes methotrexate (n=1); azathioprine, alone (n=1) or in combination with prednisone (n=2); prednisone, alone (n=1) or in combination with HCQ (n=2). Values are median [range] unless stated otherwise.

### Monocyte Isolation and RNA Isolation

Monocytes were isolated from peripheral blood mononuclear cells by magnetic-activated cell sorting using the CD14^+^ isolation kit (Miltenyi Biotec) according to the manufacturer’s instructions. The frequency of monocytes subsets and the purity of the isolated monocytes was measured by flow-cytometry to insure consistent results (**Supplementary Table 1**). Monocytes subsets were defined as the following: classical monocytes (CD14^+^CD16^-^), intermediate monocytes (CD14^+^CD16^+^), and non-classical monocytes (CD14^-^CD16^+^) (**Supplementary Figure 1**). The purity of the isolated samples was (median [range]) 96% [89–99%], and there were no significant differences in cell purity between any of the groups. Cells were lysed in RLTplus buffer (Qiagen) supplemented with 1% beta-mercaptoethanol and total RNA was purified using AllPrep Universal Kit (Qiagen), according to the manufacturer’s instructions. RNA concentration was assessed with Qubit RNA Kit (Thermo Fisher Scientific) and RNA integrity was measured by capillary electrophoresis using the RNA 6000 Nano Kit (Agint Technologies); all samples had RIN-score >7.0.

### RNA Sequencing and Data Analysis

RNA sequencing was performed at the Beijing genomics institute, using an Illumina HiSeq 4000 sequencer (Illumina) following the standard manufacturer’s protocols. About 20 million 100bp paired-end reads were generated for each sample. RNA‐sequencing data analysis was performed as previously described (31).

Briefly, quality control of the reads was assessed with FastQC tool (https://www.bioinformatics.babraham.ac.uk/projects/fastqc/), and all samples passed the quality check. Next, reads were aligned to the human genome using STAR aligner (32). The Python package HTSeq (33) was used to calculate the read counts for each annotated gene. Differentially expressed genes (DEGs) were identified using Bioconductor/R package DESeq2 (34); Wald’s test was used to identify DEGs in each pair-wise comparison performed between the three groups (HC, nSS, and pSS) and likelihood ratio test to identify DEGs considering multiple groups. P-values were corrected using a false discovery rate (FDR) of 5% according to the Benjamini and Hochberg method. Differences in gene expression with a corrected p-value<0.05 were considered differentially expressed. Variance stabilizing transformation was applied to obtain normalized gene counts (variance stabilized data), which were used for subsequent analyses.

### Gene Set Enrichment Analysis

Gene set enrichment analysis was conducted with Broad Institute software (35), to explore the contribution of each monocyte subset to the transcriptomic profile identified in monocytes from pSS patients. Thousand random permutations were performed to calculate the normalized enrichment score and FDR-corrected p-value. Gene sets were considered significantly enriched with a corrected p-value<0.05. Gene sets were obtained from a publicly available microarray dataset which identified the representative gene signature of each monocyte subsets (classical, intermediate, and non-classical monocytes) (10).

### Weighted Gene Co-Expression Network Analysis

To study the inter-dependence between genes and their interactions, a gene co-expression network was performed using weighted gene co-expression network analysis (WGCNA) Bioconductor/R package (36). Gene co-expression networks were constructed using all differentially expressed genes (corrected p<0.05) in both comparisons, nSS or pSS *vs.* HC (**Supplementary Figure 2**). Networks were defined by first performing an unsigned pairwise spearman’s correlation between genes and subsequently transforming the co-expression similarities into an adjacency matrix and scaling the adjacency matrix to achieve a scale-free topology (scaling power = 9, selected to have a network that fits the scale-free topology criterion). The identified networks were labelled using a conventional color scheme (blue, brown, turquoise and yellow) and further designated as gene signatures.

### Pathway Enrichment Analysis

To understand the biological meaning of the identified signatures, pathway enrichment analysis was performed in the different sets of genes using the compareCluster function in the ReactomePA package (37) to compare and plot the pathways enriched in the different signatures.

### Identification of Key Signatures and Hub-Genes Associated With Clinical Traits

For each signature, the expression profile was summarized by the first principal component of the signature expression level to calculate the module eigengene expression, which represents the average expression level of the genes within the signature. Next, to identify signatures that were correlated with relevant clinical features of pSS patients, a module-trait association was calculated using the pearson’s correlation coefficient between the module eigengene and the clinical traits.

To gain further insight into the signatures and the genes that have the closest relationship with the clinical features, an intramodular analysis was performed. Module membership defined as the degree of correlation between the gene and the signature, and gene significance calculated as the absolute value of the association between the gene expression profile and each clinical trait were used. Correlation with a r value higher than 0.3 and a p-value<0.05 were plotted and selected for hub-gene identification. Hub-genes were identified in the brown, yellow and turquoise signature and defined by a module membership higher than 0.8 and a gene significance higher than 0.4. Next, hierarchical cluster analysis of the hub-gene expression based on Ward’s method with Euclidian distance was performed using the MetaboAnalyst5.0 online software (http://www.metaboanalyst.ca/) to identify patients with a similar inflammatory profile.

The IFN-score was calculated using the gene expression of five IFN-induced genes (*IFI44L*, *IFI44*, *IFIT3*, *LY6E*, and *MX1*), after z-score normalization, as previously described (22).

### TNF-α Production Upon Toll Like Receptor Activation

As functional annotation of the turquoise signature revealed enrichment in genes involved in TLR-signaling we set out to confirm these alterations on a functional level. For this purpose, whole blood was diluted 1:1 in RPMI-1640 medium with 1% L-glutamine (both from Thermo Fisher Scientific) and stimulated with different TLR ligands (**Supplementary Table 2**). 1h after stimulation, 10μg/mL of Brefeldin A (Sigma) was added. After a total of 6h of stimulation cells were stained with the extracellular antibodies and after washing, fixation and permeabilization with FIX&PERM (Thermo Fisher Scientific), cells were stained with anti-TNF-α (**Supplementary Table 1**). Data acquisition was performed using a BD LSRFortessa (BD Biosciences) and data were analyzed using FlowJo software (Tree Star).

### *In Vitro* Monocyte Stimulation and IFNα/β Receptor Blockade

Monocytes from HC were isolated as previously described, and treated with serum of HC or pSS patients (20% v/v) for 20h, either alone or in the presence of TLR4 ligand (0.1μg/mL of LPS; Invivogen) for the last 17h of culture. Simultaneously, monocytes were pre-incubated for 1h at 37°C with 5μg/ml of either anti-IFNα/βR2 (clone MMHAR-2; PBL Assay Science) or its respective isotype control (IgG2a, Thermo Fisher Scientific), and then treated with serum of pSS patients (20% v/v) for 24h. Supernatants were harvested and cells lysed in RLTplus buffer (Qiagen). RNA isolation was performed as previously described. Quantitative-PCR reactions were performed using SYBR Select Master Mix (Applied Biosystems) on the Quantstudio 12k system (Thermo Fisher Scientific). Relative gene expression levels were normalized to the geometric mean expression of two housekeeping genes: *B2M* and *ACTB* (**Supplementary Table 3**). The relative fold change (FC) of each sample was calculated in relation to the average ΔCt of the HC group or to the ΔCt of the respective isotype condition (reference), according to the formula FC = 2^−ΔΔCt^, where ΔΔCt = ΔCt sample —ΔCt reference. TNF-α levels were measured in the supernatants by ELISA (Diaclone), following the manufacturer’s instructions.

### Statistics

Differences in gene expression with a corrected p-value<0.05 were considered statistically significant. Kruskal-Wallis H test was used to analyze the differences between the nSS, pSS, and HC groups and between patient clusters. Fisher’s exact test was used to compare categorical variables. Differences between pSS patients and HC were assessed using Mann-Whitney U-test. Changes observed after IFNα/βR2 blocking were analyzed using Wilcoxon matched-pairs signed rank test. Statistical analyses and data visualization were performed using Python and R, Graphpad Prism (GraphPad Software), and MetaboAnalyst 5.0 (38). Differences were considered to be statistically significant at p<0.05.

## Results

### Transcriptome of pSS Monocytes Is Enriched for Gene Expression Profiles Associated With Intermediate and Non-Classical Monocytes

**Figure 1:**
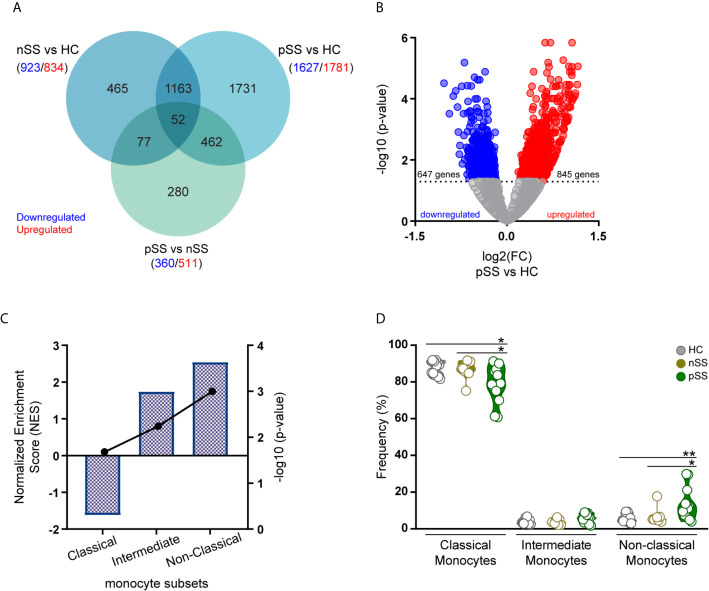
Transcriptomic profile of monocytes from pSS patients is enriched for genes associated with intermediate and non-classical monocytes. RNA sequencing of peripheral blood isolated monocytes of nSS and pSS patients and HC was performed and differentially expressed genes (DEGs) were identified. Venn diagram shows the overlap of the DEGs between the different comparisons with a nominal p-value < 0.05, downregulated (blue) or upregulated (red) genes are indicated for each comparison **(A)**. The relationship between the fold change (log2) and the corrected p-value (-log10) of the DEGs in pSS *vs.* HC is displayed. DEGs, with a corrected p-value < 0.05, downregulated (blue) or upregulated (red) in pSS-monocytes are indicated **(B)**. Gene-set enrichment analysis was performed comparing the DEGs identified in pSS patients to the molecular signature of the different monocyte subsets. Columns and left y-axis show the normalized enrichment score, black connected dots and right y-axis displays the FDR-corrected p-value (-log10) **(C)**. Frequency of monocyte subsets assessed by flow-cytometry in HC, nSS and pSS patients after cell isolation is shown **(D)**. * and ** represent p < 0.05 and p < 0.01, respectively.

To better understand the contribution of monocytes to the development of pSS and to identify monocyte-specific processes dysregulated in pSS, we performed RNA-sequencing profile of circulating monocytes of pSS and nSS patients compared to HC (**Table 1**). Differential expression analysis revealed that among the studied groups, 6.036 genes were differentially expressed with a nominal p-value ≤ 0.05, of which 3.126 were upregulated and 2.910 downregulated. The majority of the differentially expressed genes (3.408 DEGs) were found in the pSS *vs.* HC comparison, representing 56% of all the DEGs, and out of these 1.731 were uniquely differentially between pSS and HC. In addition, 29% off all DEGs (1.757 genes) were differentially expressed in nSS patients *vs.* HC, and only 465 (26%) were exclusively found differentially expressed between nSS and HC. Furthermore, in the comparison between pSS and nSS, out of the 871 DEGs only a small number of genes (280/32%) was uniquely differential between pSS *vs.* nSS (**Figure 1A**). Overall, the transcriptomic profile of pSS monocytes revealed clearly distinct expression profiles compared to HC monocytes, but relatively similar profiles to nSS monocytes. To identify the most robust and consistently altered genes, those differentially expressed with an FDR corrected p-value ≤ 0.05 and with at least moderately high expression levels (base mean expression >100) were selected for further analysis (**Supplementary Figure 2**).

To gain further insights into the molecular signature of pSS monocytes, we performed gene set enrichment analysis to evaluate the contribution of each monocyte subset to the transcriptional profile found in pSS monocytes. To test this, we compared the DEGs in pSS patients that met the selection criteria (**Figure 1B**) with publicly available gene expression profiles of the three different monocyte subsets (10). Enrichment analysis revealed that genes highly expressed in intermediate and non-classical monocytes were significantly enriched in the transcriptional profile of pSS monocytes (**Figure 1C**). In line with this, flow-cytometry analysis of the monocyte subsets confirmed that the non-classical monocyte subset was increased in the circulation of pSS patients when compared with nSS patients and HC (**Figure 1D**). Thus, the transcriptomic alterations found in pSS monocytes are consistent with an increased frequency of non-classical monocytes.

### Network Analysis Identifies Signatures of Co-Expressed Genes Associated With Systemic Inflammatory Parameters

**Figure 2:**
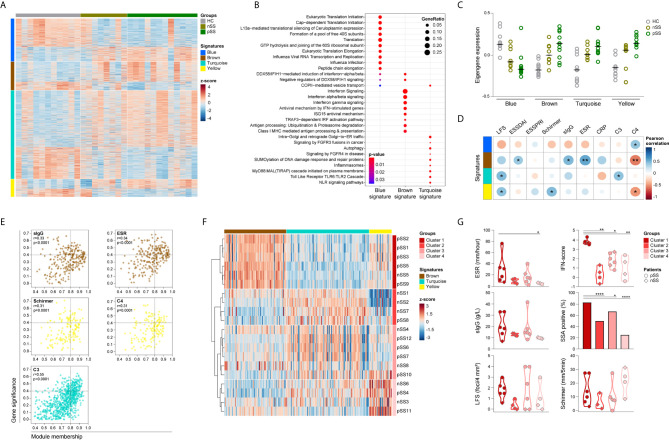
Identification and functional characterization of pSS-monocytes signatures reveals associations with markers of local and systemic inflammation. Weighted gene co-expression network analysis was performed to identify signatures of co-expressed genes and their respective functional annotation. Heatmap visualization of the 1500 DEGs across the 4 identified signatures (blue, brown, turquoise and yellow; rows) and the studied groups (HC, nSS and pSS; columns) **(A)**. Reactome pathway enrichment analysis was used for functional annotation of the signatures, there was no result for the yellow signature. Dot-size depicts the fraction of the genes within the pathway that is enriched, color indicates the statistical significance of the enrichment **(B)**. Eigengene expression (first principal component of the module expression level) for each signature is depicted for HC, nSS and pSS patients **(C)**. Correlation matrix of the module eigengenes and the clinical traits is depicted. Signatures are shown in rows and the clinical traits in columns. Dot-size and color indicates the Pearson correlation coefficient, p-values are shown inside the dots; **(D)**. Intramodular analysis identifies hub-genes related with markers of systemic and local inflammation. Scatter plots of gene significance for the selected clinical trait versus the module membership per signature are depicted. Each dot represents one specific gene within a signature. Hub-genes are defined by a module membership of >0.8 and a gene significance of >0.4. Signatures can be identified by the corresponding color of the graph **(E)**. nSS and pSS patient were clustered using hierarchical clustering of the selected hub-genes using Euclidean distance and Ward’s method. Hub-genes of each signature are shown in columns and the patient clusters are indicated in rows **(F)**. Violin plots depicts the expression of selected clinical parameters across the established patient clusters **(G)**. *, ** and **** represent p < 0.05, p < 0.01, and p < 0.0001, respectively.

To further analyze the functional consequences of the transcriptomic changes in pSS monocytes, we used weighted gene co-expression network analysis (WGCNA) to construct gene-correlation networks and identify signatures of co-expressed genes with pSS-associated biological functions. Network analysis using the genes that were differential between any of the three groups compared (**Supplementary Figure 2**) identified 4 gene signatures: blue (358 genes), brown (239 genes), turquoise (737 genes), and yellow (148 genes), each with distinct expression patterns (**Figure 2A** and **Supplementary Table 4**). 18 of the DEGs were not allocated to any of the previous signatures and therefore excluded from further analysis.

Functional annotation indicated that the blue signature was associated with translation mechanisms, the brown signature with IFN-signaling, and the turquoise signature with TLR signaling (**Figure 2B**). No pathways were enriched for the yellow signature. Next, we further characterized the identified signatures, by assessing the module eigengene expression, given by the first principal component of the signature expression level, in HC, nSS, and pSS patients. Overall, the expression of genes from the blue signature was decreased in nSS and pSS patients and contains a large number of ribosomal protein genes, and a range of initiation factors and RNA polymerase subunit genes. Conversely, the expression of the brown, turquoise, and yellow signatures was largely increased in both patient groups, represented by an increased eigengene expression. The expression patterns of all four gene signatures in the nSS group were in general intermediate between the HC and pSS groups (**Figure 2C**).

### Identified Gene Signatures Are Associated With Systemic Inflammatory Parameters

To better understand the biological relevance of the different signatures for the disease, we next assessed the correlation between clinical traits (**Table 1**) and the eigengene expression in each signature. Eigengene expression is an ideal parameter to correlate with the clinical traits, since it represents the first principal component of the co-expressed gene network and thus accounts for most of the variance in gene expression within the signature. Genes from the brown signature had the most correlations with the clinical traits, including the strongest positive correlation with ESR (r = 0.51, p = 0.003), and with ESSDAI (r = 0.37, p = 0.042). In addition, the lymphocytic focus score (LFS), which reflects salivary gland inflammation, moderately correlated with the turquoise and yellow signatures (r = 0.43, p = 0.015 and r = 0.40, p = 0.024, respectively), whereas only the yellow signature moderately correlated with Schirmer test, a measure for lacrimal gland function (r = 0.44, p = 0.013). Of note, moderate negative correlations were found with C4 levels for the brown and yellow signatures (r = -0.51, p = 0.003 and r = -0.43, p = 0.016, respectively) (**Figure 2D**).

Next, we performed an intramodular analysis to seek out the biological significance of each gene within the respective signature for the clinical features that correlated with the signature expression (**Figure 2D**). The genes within the brown, and turquoise signatures significantly correlated with sIgG, ESR, and complement C3 levels, parameters usually associated with systemic inflammation. Likewise, the genes within the yellow signature correlated with complement C4 levels and with Schirmer test, indicative of lacrimal gland dysfunction (**Figure 2E**). No correlations were observed for LFS and ESSDAI. Genes with a module membership (a higher number indicates stronger correlation of the gene with the other genes in the signature) higher than 0.8 and a gene significance (a higher number indicates stronger correlation with the selected clinical trait) higher than 0.4 were selected as hub-genes for each signature independently. Analysis of the brown signature revealed 43 hub-genes with gene significance for sIgG, and 68 hub-genes with gene significance for ESR. Out of these, 31 hub-genes were found with gene significance for both parameters (**Figure 2E** and **Supplementary Table 5**). Within the yellow signature, 16 hub-genes were identified with gene significance for Schirmer test and 13 were found with gene significance for C4 levels, of these 7 hub-genes were identified with gene significance for both the Schirmer test and C4 levels (**Figure 2E** and **Supplementary Table 6**). In the turquoise signature, analysis of gene significance for C3 complement levels yielded 107 hub-genes (**Figure 2E** and **Supplementary Table 7**). Thus, the molecular signatures of circulating monocytes from pSS patients seems to reflect the ongoing systemic inflammation as result of local damage.

Given the fact that hub-genes are highly associated with the clinical features of pSS, we next investigated whether the hub-gene expression could be used to identify patients with a similar inflammatory profile. Unsupervised hierarchical cluster of the selected hub-genes (**Supplementary Tables 5**–**7**) identified four distinct clusters of nSS and pSS patients. Cluster 1 mainly included patients diagnosed as pSS patients (5 out of 6), and all patients were characterized by an increased expression of the brown signature (**Figure 2F** and **Supplementary Figure 3A**). As a matter of fact, patients in cluster 1 showed the highest IFN-score compared to the patients included in other clusters (**Figure 2G**). Clustering analysis identified a group of patients included in cluster 2 with a pronounced downregulation of the yellow signature hub-genes, and a trend towards a lower LFS, ESR, sIgG and IFN score (**Figure 2G** and **Supplementary Figure 3A**). Patients in cluster 3 were characterized by an overexpression of the turquoise signature and displayed an intermediate profile for ESR, IFN-score and frequency of anti-SSA antibodies, as compared with patients in cluster 1 and 2 (**Figure 2G** and **Supplementary Figure 3A**). Patients in cluster 4 did not display a specific signature profile (**Supplementary Figures 3A, B**). Furthermore, comparison of systemic autoimmune and inflammatory parameters between clusters showed that patients in cluster 1 have an increased frequency of anti-SSA antibodies and presented with higher ESR. Moreover, a trend towards a higher sIgG and lower C3 and C4 levels was observed in these patients compared to the patients in the other clusters (**Figure 2G** and **Supplementary Figure 3B**). Finally, patients in cluster 1 have higher frequency of non-classical monocytes compared to the patients in cluster 3 and 4 (**Supplementary Figure 3C**).

### pSS-Monocyte Produce More TNF-α Upon Toll-Like Receptor Stimulation

**Figure 3:**
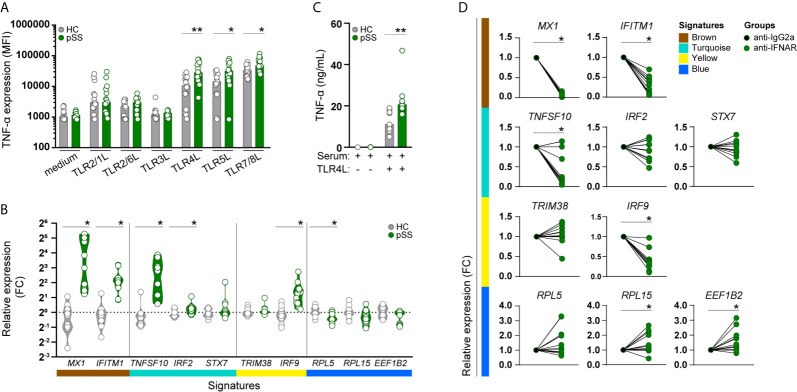
Monocytes from pSS patients produce more TNF-α upon TLR stimulation, and its transcriptomic signature is mimicked in HC monocytes by pSS serum and is partially prevented by IFNα/β receptor blockade. TNF-α cytokine production (given by the median fluorescence intensity, MFI) was assessed by flow-cytometry in CD14^+^ monocytes, after whole blood stimulation with TLR ligands (HC n= 15, pSS n= 15) **(A)**. The effect of serum from HC or pSS patients (20% v/v; HC n= 12, pSS n= 11) treatment on monocytes from HC was assessed after 20h by qRT-PCR. Changes in the expression of the hallmarks of each signature were assessed **(B)**. Monocytes from HC were pre-treated with serum from HC or pSS patients (20% v/v; HC n= 11, pSS n= 10) for 3h and then left untreated or stimulated with TLR4 ligand (0.1μg/mL of LPS) for a total of 20h. TNF-α secretion was measured by ELISA **(C)**. Monocytes from HC were pre-treated for 1h with an isotype control (IgG2a) or an anti-IFNα/βR2 blocking antibody and subsequently treated with pSS serum (20% v/v; n=7) for 20h. Changes in the expression of the hallmark genes representing the pSS-monocyte signatures were assessed by qRT-PCR **(D)**. * and ** represent p < 0.05, and p < 0.01, respectively.

Considering that the functional annotation of the turquoise signature revealed enrichment in genes involved in TLR-signaling, we next assessed whether the altered transcriptional profile of pSS-monocytes could impact monocyte activation. To confirm this, whole-blood from pSS patients and HC was stimulated with a broad panel of toll-like receptor ligands and the intracellular expression of TNF-α was evaluated by flow cytometry (**Supplementary Figure 4**). Interestingly, the intracellular TNF-α expression in monocytes of pSS patients, as determined by the median fluorescence intensity (MFI), was significantly increased upon activation with TLR4, TLR5, and TLR7/8 ligands compared to HC monocytes (**Figure 3A**). We did not observe differences in TNF-α expression between HC and pSS monocytes upon TLR2/1, TLR2/6, and TRL3 activation (**Figure 3A**).

### Exposure of Healthy Control Monocytes to Serum of pSS Patients Mimics the pSS-Monocyte Gene Signature

As the molecular signatures of monocytes from pSS patients and the expression of their hub-genes were mainly associated with systemic inflammatory markers, we sought to confirm whether the transcriptomic changes found in pSS-monocytes could be induced in monocytes from healthy individuals by sera of pSS patients. To this end, we selected hallmark genes to represent each signature, and ultimately the transcriptomic profile of pSS monocytes, by selecting those hub-genes with the highest degree of correlation with each signature (high module membership), the highest biological relevance (high gene significance) and highest fold-change of expression differences between pSS and HC (**Supplementary Table 8**). Treatment with serum from pSS patients upregulated both hallmark genes of the brown signature (*MX1*, *IFITM1*), two of the three turquoise hallmark genes (*TNFSF10*, *IRF2*), one of the two yellow signature hallmark genes (*IRF9*), and downregulated one of the three blue signature hallmark genes (*RPL5*) (**Figure 3B**). In addition, we tested whether the inflammatory mediators present in the serum of pSS patients could prime healthy monocytes for higher TNF-α production upon TLR activation. Due to limited amount of serum, we were only able to test this upon TLR4 activation. We observed that the changes induced by serum from pSS patients led to a significantly higher TNF-α production upon TLR4 stimulation (**Figure 3C**).

### Type-I IFNs Importantly Mediate Effects of pSS Serum on Signature Hallmark Gene Expression

As type-I IFNs are considered to be crucial mediators of pSS pathogenesis (39), we tested whether the transcriptomic alterations were mediated by type I IFNs. Interferon-α/β receptor (IFNAR) blockade completely abrogated the pSS-serum effect on the brown gene signature, and also reverted the pSS-serum effects on *TNFSF10* and *IRF9* expression, hallmark genes of the turquoise and yellow signature, respectively. In addition, *RPL5* expression, hallmark of the blue signature, was upregulated upon IFNAR blockade, although not statistically significant. Even though the expression of *RPL15* and *EEF1B2*, hallmarks of the blue signature, were not significantly downregulated upon treatment with serum from pSS patients, IFNAR blockade significantly increased the expression of both genes. (**Figure 3D**). Thus, type-I IFNs importantly drive the observed effects of pSS serum on the identified signature hallmark genes in monocytes.

## Discussion

We here identified a large number of transcriptomic differences in circulating monocytes between patients with pSS and nSS and HCs. In addition, we observed an enrichment in the non-classical monocyte subset in pSS patients, and identified gene signatures in pSS monocytes which indicated dysregulation in translation processes, IFN-signaling, and TLR signaling. Furthermore, we show that the hub-genes of the identified gene signatures correlate with systemic and local inflammatory markers, indicating that the transcriptional dysregulation of pSS monocytes reflects ongoing inflammation. Interestingly, we demonstrated that unsupervised hierarchical cluster of the hub-gene expression identified four distinct clusters of nSS and pSS patients with different inflammatory profiles. Finally, we confirmed that the transcriptomic aberrations of pSS monocytes can be induced, at least in part, by mediators present in the circulation of pSS patients, including type-I IFNs. Thus, the systemic inflammatory profile of pSS patients play an important role in shaping the monocyte transcriptomic profile and ultimately their function.

The current study shows that pSS monocytes are skewed to a pro-inflammatory profile, with elevated frequency of non-classical monocytes, consistent with previous reports (3, 17). This increased frequency might be related with the differentiation of classical monocytes into intermediate/non-classical monocytes sustained by the inflammatory environment, as previously described in patients with SLE and rheumatoid arthritis (40, 41). In addition, CD16^+^ monocytes more efficiently produce pro-inflammatory cytokines, including C-C Motif Chemokine Ligand 3 (CCL3), TNF-α and IL-6 (42, 43), known to be increased in pSS (44–46), thus contributing to the maintenance of the inflammatory environment. As such, these data suggest that the increased numbers of non-classical monocytes enhance the inflammatory environment sustaining the differentiation of non-classical monocytes and therefore pSS immunopathology.

Using a gene co-expression network analysis, we have identified three new molecular gene signatures (blue, turquoise, yellow), which were not identified previously using differential expression analysis (25). In addition, we also identify differentially expressed genes directly related to the IFN-signature, as part of the brown signature, in line with previously reports (22, 25). Association of the brown signature with ESSDAI, complement consumption and with sIgG, a marker of B cell hyperactivity in pSS, indicates that the transcriptomic alterations induced by type I IFN contributes to enhance B cell activation and disease activity. In fact, IFN stimulation was shown to increase BAFF production by monocytes (22, 28), a key cytokine for B cell survival and activation (47), and CXCL13 production, crucial for ectopic lymphoid structures organization (48, 49), which can be found in pSS patients (50, 51). Moreover, non-classical monocytes from SLE patients, a type-I IFN disease, more efficiently promote B cell differentiation towards memory and plasma cells and IgG production (15). As such, the modulation of monocytes signature by type I IFN, is an important mechanism to promote B cell hyperactivity and local inflammation.

Functional annotation of the newly identified signatures revealed that the blue gene signature is primarily related with the cellular processes of translation, yet other relevant functions in innate immunity, including regulation of cytokine signaling, have been reported (52). Downregulation of ribosomal protein genes has been described in LPS-activated monocyte-derived dendritic cells and in peripheral blood mononuclear cells stimulated with type-I IFN (53) to act as a regulatory mechanism of cell activation. The turquoise gene signature importantly shows enrichment for TLR activation and signaling and is associated with the lymphocytic infiltration of the salivary glands (LFS). Thus, indicating that monocytes from pSS patients display an activated profile, in line with previous reports (20, 28), reinforcing the relevance of the altered monocyte function to the salivary glands’ inflammation. Consistent with this, it was shown that CD16^+^ monocytes can differentiate into dendritic cells with a phenotype similar to those observed in salivary gland infiltrates in pSS patients (3). Functional annotation of the genes within the yellow signature, did not indicate any specific function, possibly due to the size of this signature, as it includes in total 128 protein coding genes, limiting the identification of share processed by these genes. Nonetheless, this signature was associated with LFS and Schirmer test, suggesting a potential link of these set of genes with immune cell infiltration, and damage of the exocrine glands. As such, further research is needed to shed more light on the function of the yellow signature and confirm the role of the monocyte transcriptome in the regulation of local inflammation.

To the best of our knowledge, this is the first WGCNA analysis of RNA-sequencing data from monocytes of nSS and pSS patients which identifies hub-genes associated with systemic and local inflammatory markers. Although nSS patients may share severe objective dryness, and occasionally present with single systemic features similar to pSS patients, yet not compatible with generalized autoimmune disease, this group of patients is often poorly studied and difficult to characterize. Given the high-level of gene significance of the identified hub-genes with the clinical features of pSS we propose that their gene expression would allow the identification of patients, both nSS and pSS, with similar systemic or local inflammatory profile. Our in-depth characterization of the monocyte transcriptome demonstrates that the dysregulated mechanisms found in pSS monocytes are present in some nSS patients. As such, patient clustering based on different molecular signatures holds promise for a better characterization of sicca patients and implementation of novel therapeutic targets. We here provide proof of concept for further exploration of the transcriptome of nSS patients, as some patients share a molecular signature with pSS patients whereas the signs of local and peripheric autoimmunity are not yet present. Nevertheless, follow up studies of both nSS and pSS patients and longitudinal analysis in larger cohorts are needed to shed light on the role of the transcriptional signatures of circulating cells, and on its value for the implementation of new therapeutic options.

Transcriptomic analysis of pSS patients’ monocytes suggests that these cells have an altered activation status, which was confirmed by the increased production of TNF-α by pSS monocytes. Moreover, increased NF-kB activation was previously observed in pSS-monocytes (20), which is in line with our findings, since NF-kB has a central role in the production of pro-inflammatory cytokines, including TNF-α (54). TNF-α is a potent pro-inflammatory cytokine known to be increased in tear fluid and saliva of pSS patients (55, 56), and has been strongly implicated in the apoptosis of salivary gland cells (57), causing secretory dysfunction (58, 59), contributing to the salivary gland destruction. This enhanced TNF-α production by monocytes of pSS patients could be related with the increased frequency of non-classical monocytes which we observed, as they are known to produce high amounts of TNF-α (16, 60). Although the results of anti-TNF-α treatment in pSS patients were discouraging, this cytokine does seem to play an important role in pSS immunopathology.

Furthermore, the tight association of the transcriptomic signatures from monocytes of pSS patients with the systemic inflammatory markers suggests a contribution of these mediators to reshape the monocyte transcriptome towards an inflammatory phenotype. Our results support this concept, demonstrating that serum from pSS patients modulates the transcriptome of healthy controls monocytes resembling the profile found in pSS patients. Moreover, the alterations induced by pSS-serum primed the monocytes to an increase production of TNF-α upon TLR stimulation, similar to what was observed in pSS patients after ex-vivo whole blood stimulation. Given the cross-regulation of TNF-α and type I IFN (61), and the fact that type-I IFN priming enhances TNF-α production upon stimulation (62) we hypothesize that the increased TNF-α production observed in pSS patients upon stimulation could be in part, a consequence of an altered transcriptomic profile induced by type I IFNs. In fact, IFNAR blockade partially abrogated the effect of pSS-serum in HC-monocytes, suggesting that the transcriptomic reshape of pSS monocytes consists of IFN-dependent and independent mechanisms. The IFN-dependent mechanisms was observed not only in the brown signature, which mainly reflects the IFN-signaling genes, but also in the hallmarks of the blue signature. The genes contained in this signature are involved in the regulation of gene and protein expression (63, 64) and are known to be downregulated upon stimulation as a regulatory mechanism of cell activation. As monocytes from pSS patients show a reduced expression of this signature, and IFNAR blockade restores it, our results suggest that the translation processes dysregulated in pSS are a consequence of monocyte activation by type-I IFNs. This may induce a loop of inflammation, which can activate other immune cells and favors pSS immunopathology.

A possible limitation of our study is represented by the sample size of the cohort used for RNA-sequencing and WGCNA analyses. Nevertheless, we were able to successfully replicate published findings (3, 20, 22, 25), and we used two independent cohorts of pSS patients and HC to experimentally validate our results. Future studies with integrated analysis of different multi-omics data could shed light on how the identified hub-genes and associated hub-signatures can affect pSS immunopathology.

Taken together, we show that monocytes from pSS patients have a transcriptome enriched in gene expression profiles associated with intermediate and non-classical monocytes subsets. As well, pSS monocytes show dysregulation in gene signatures involved in processes related with translation, IFN-signaling and TLR-signaling. In addition, the correlation of hub-genes with the peripheral and local inflammatory mediators indicates that the transcriptional dysregulation of pSS monocytes results from their inflammatory environment. Finally, we confirm that the pSS monocyte transcriptome is partially induced by circulating mediators present in serum of pSS patients, and that IFNAR blockade in part prevents these alterations. In view of the important role of monocytes in the activation of innate and adaptive immunity, our data supports the concept that targeting the molecular mechanism underlying monocyte activation, including type-I IFNs holds promise for halting the immunopathology of Sjögren’s Syndrome.

## Data Availability Statement

The RNA sequencing datasets generated for this study can be found in NCBI’s Gene Expression Omnibus (GEO) and are accessible through GEO Series accession number GSE173670. The raw data supporting the conclusions of this article will be made available by the authors, without undue reservation, to any qualified researcher. 

## Ethics Statement

The studies involving human participants were reviewed and approved by the board of medical ethics committee of the University Medical Center Utrecht (METC no. 13-697). The patients/participants provided their written informed consent to participate in this study.

## Author Contributions

AL, MH, SB, AK, TR, and JR were involved in conception and design of the study. AL, CB, MH, SB, AH, and AK were involved in data acquisition. AL, CB, MH, AP, AK, TR, and JR were involved in data analysis and interpretation. AL drafted the manuscript. All authors contributed to the article and approved the submitted version.

## Funding

AL was supported by a Ph.D. grant from the Portuguese national funding agency for science, research and technology: Fundação para a Ciência e a Tecnologia (SFRH/BD/116082/2016). MH was supported by the Dutch Arthritis Society (Grant no. 14-2-301). AP was funded by the Netherlands Organization for Scientific Research (NWO; Grant nr 016.Veni.178.027). TR received research funding from GlaxoSmithKline for his work on Sjögren’s syndrome as part of the GSK Immune Catalyst Program. The funding sources had no role in study design; data collection, analysis, and interpretation; writing the report; or in the decision to submit the article for publication.

## Conflict of Interest

TR was a principal investigator in the immune catalyst program of GlaxoSmithKline, which was an independent research program. He did not receive any financial support other than the research funding for the current project. Currently, TR is an employee of Abbvie where he holds stock. TR had no part in the design and interpretation of the study results after he started at Abbvie.

The remaining authors declare that the research was conducted in the absence of any commercial or financial relationships that could be construed as a potential conflict of interest.

## Publisher’s Note

All claims expressed in this article are solely those of the authors and do not necessarily represent those of their affiliated organizations, or those of the publisher, the editors and the reviewers. Any product that may be evaluated in this article, or claim that may be made by its manufacturer, is not guaranteed or endorsed by the publisher.
