# Supplementary material for: The Transcriptomic Profile of Monocytes from Patients With Sjögren’s Syndrome Is Associated With Inflammatory Parameters and Is Mimicked by Circulating Mediators
Source: Front Immunol. 2021 Aug 3;12:701656. doi: 10.3389/fimmu.2021.701656 (PMC8368727; doi:10.3389/fimmu.2021.701656)
Supplement: Supplementary file 1 [file DataSheet_1.pdf]

Supplementary Material

1 Supplementary Figures and Tables

1.1 Supplementary Figures

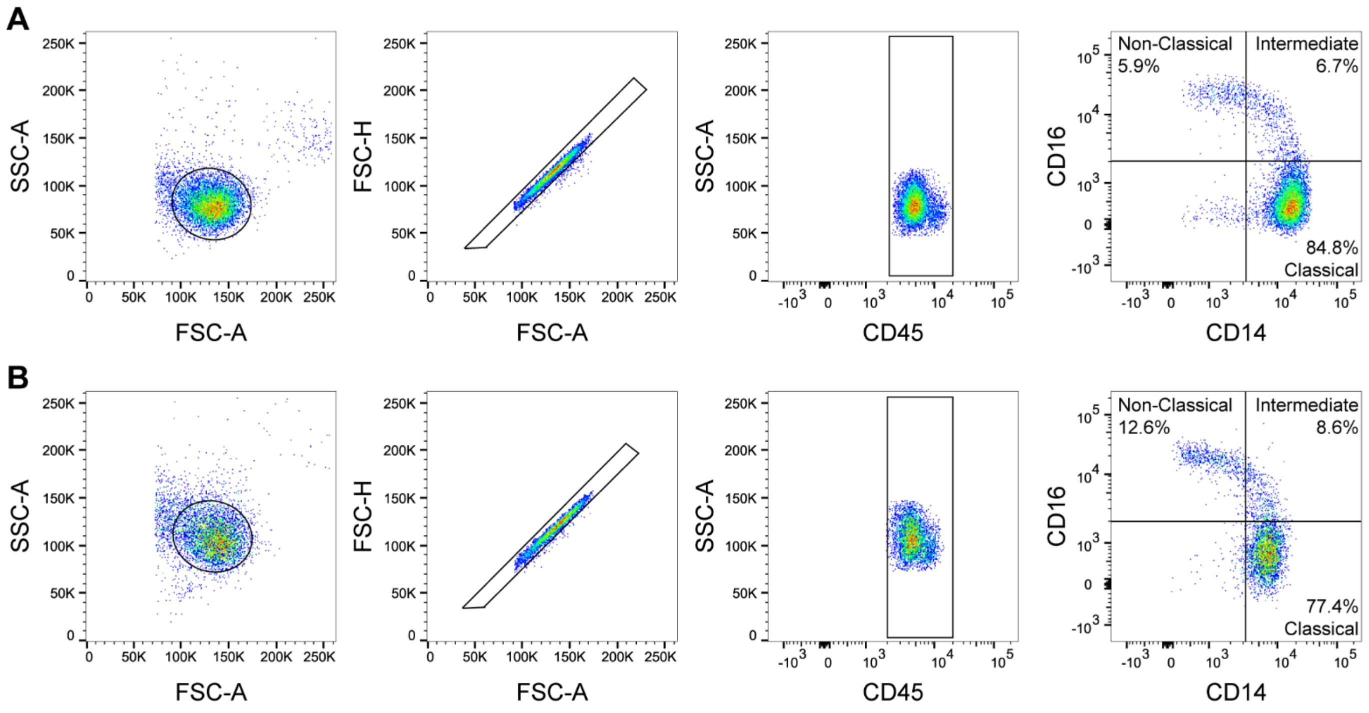

Supplementary Figure 1. Analysis strategy of monocyte subsets

Representative flow cytometry gating strategy analysis of the different monocyte subsets: classical ($CD14^+/CD16^-$), intermediate ($CD14^+/CD16^+$) and non-classical ($CD14^-/CD16^+$) monocytes in healthy controls (A) and primary Sjögren's syndrome patients (B).

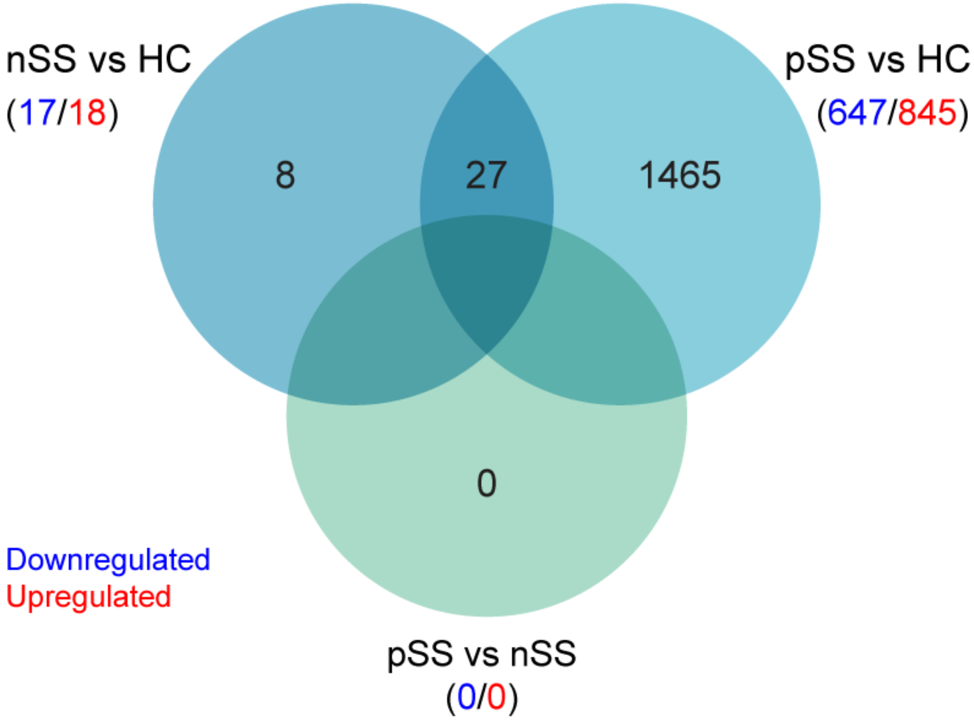

Supplementary Figure 2. Monocytes from pSS patients are transcriptionally different from HC monocytes

RNA sequencing of peripheral blood isolated monocytes of non-Sjögren's sicca (nSS), primary Sjögren's syndrome (pSS) and healthy controls (HC) was performed and the differentially expressed genes (DEG) identified. Venn diagram shows the overlap of the differential expressed genes between the different comparisons with an FDR corrected p-value $\leq$ 0.05 and base mean >100. Downregulated or upregulated genes in each comparison are indicated in blue or in red, respectively.

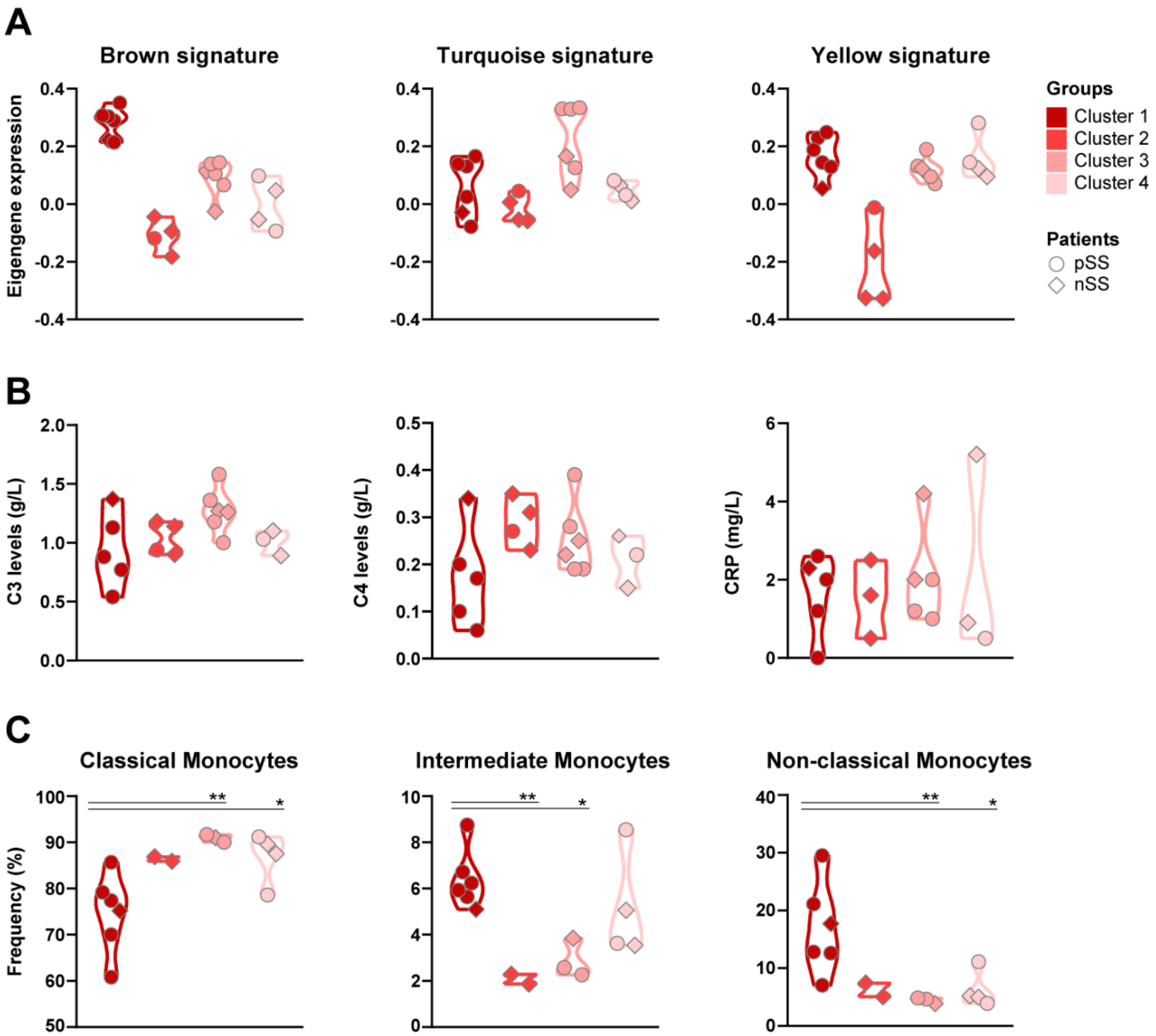

Supplementary Figure 3. Hierarchical cluster based on hub-gene expression identifies cluster of nSS and pSS patients with similar signatures

Intramodular analysis identified hub-genes, defined by a module membership > 0.8 and a gene significance > 0.4, related with systemic and local inflammatory markers. Hierarchical clustering of nSS and pSS patient based on hub-genes expression identified clusters of patients with shared features. Violin plots depicts the eigengene expression (first principal component) of each signature (A), the C3, C4 complement levels, and C-reactive protein (CRP) (B) and the frequency of the monocyte subsets across the established clusters (C).

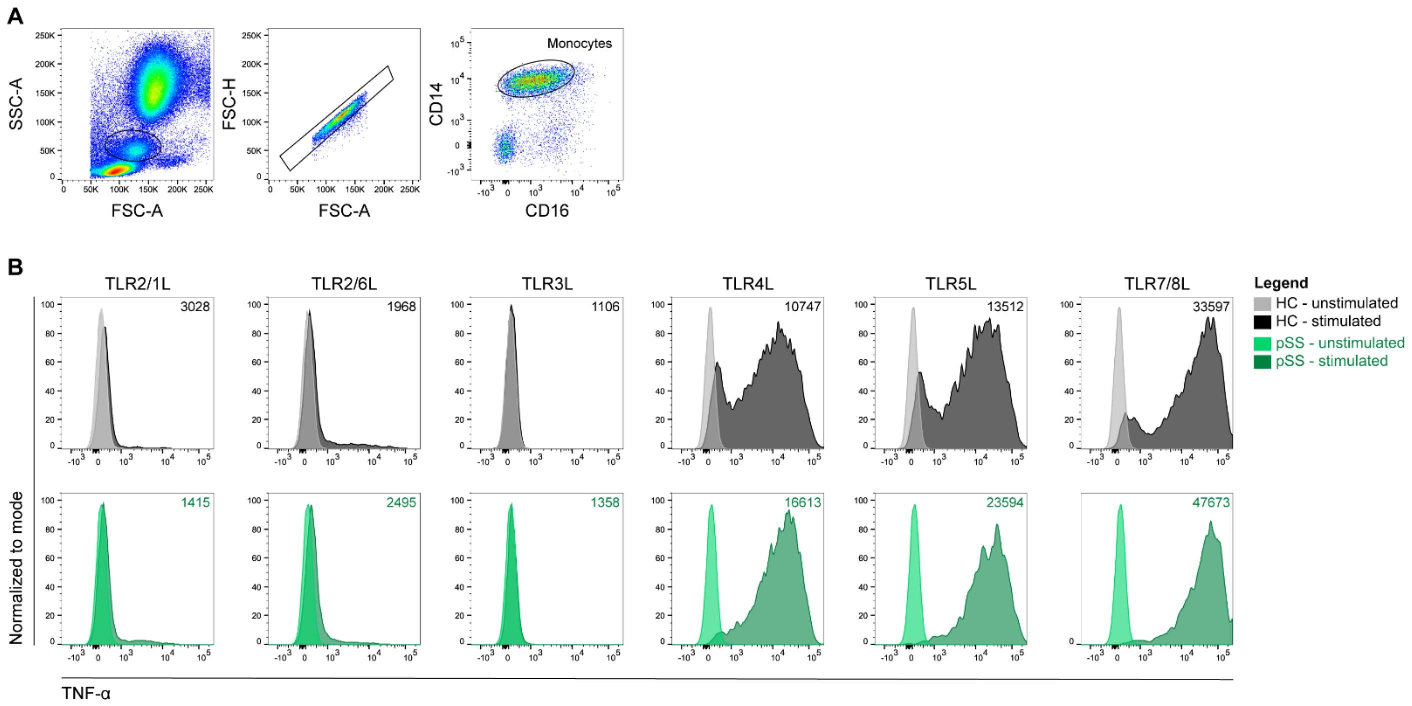

Supplementary Figure 4. Analysis strategy of intracellular TNF-α expression in CD14^{+} monocyte after TLR stimulation

Representative flow cytometry gating strategy analysis of monocyte (CD14^{+}) in healthy control (A) and intracellular TNF-α production after whole blood stimulation in monocytes of HC (black) and pSS patients (green). Unstimulated control and stimulation upon TLR ligands for 6h are depicted, as well as the median fluorescence intensity of the representative individuals (B).

1.2 Supplementary Tables

Supplementary Table 1. List of antibodies used on the flow cytometry staining

Study	Target	Label	Manufacturer	Clone
Purity	CD45	PerCP	Biolegend	HI30
	CD14	FITC	Miltenyi	TÜK4
	CD16	PE	Dako	DJ130C
Whole blood	BDCA1	APC	Thermo Fisher Scientific	L161
	CD19	BV510	Biolegend	HIB19
	HLA-DR	BV605	BD Biosciences	G46-6
	CD14	BV785	BioLegend	M5E2
	cyTNF-α	BV421	BioLegend	MAb11

Supplementary Table 2. List of the TLR ligands used for whole blood stimulation

Target	Ligand	Manufacturer	Concentration
TLR2/1	Pam3CSK4	Invivogen	5µg/mL
TLR2/6	Pam2CSK4	Invivogen	1µg/mL
TLR3	Poly(I:C)	Invivogen	25µg/mL
TLR4	LPS	Invivogen	0.1µg/mL
TLR5	Flagellin	Invivogen	2µg/mL
TLR7/8	R848	Invivogen	2µg/mL

Supplementary Table 3. Sequences of primers used for RT-qPCR

Gene	Primer forward 5’ – 3’	Primer reverse 5’ – 3’
ACTB	CATCGAGCACGGCATCGTCA	TAGCACAGCCTGGATAGCAAC
B2M	GATGAGTATGCCTGCCGTGT	TGCGGCATCTTCAAACCTCC
MX1	GCATCCCACCCTCTATTACTG	CGCACCTTCTCCTCATACTG
IFITM1	CCAGCATCCGGACACCACAG	CCCCCAGCACAGCCACCTC
TNFSF10	TGCGTGCTGATCGTGATCTTC	GCTCGTTGGTAAAGTACACGTA
IRF2	TCCATACAGGAAAGCATCAACC	CATGGCGCATCTGAAATTCGC
STX7	GGCCCAGAGGATCTCTTCTAA	ACTGTTGCCTCAATTCAGGTG
TRIM38	GTTGAAGACGTATGCCAGGG	GCTTCTGCTCCGTACATCTGTC
IRF9	AGCCACAGGAAGTTACAGAC	TAGATGAAGGTGAGCAGCAG
RPL5	GAGGCTTGTCTATCCCTCACA	GTGCTTCCGATGTACTTCTGC
RPL15	CCCACCCGGCCTGATAAAG	CACGGCGAACACGAATCCT
EEF1B2	GGTGCTCAACGATTACCTGG	ATACCAACGTAGGGCATGACA

Supplementary Table 4. List of the differential expressed genes across the identified signatures

Supplementary Table 5. List of the hub-genes identified in the brown signature

Gene	Fold change pSS vs HC	Module Membership	Gene Significance (sIgG)	Gene Significance (ESR)
DDX60	0.927	0.957	0.434	0.428
TRIM22	0.813	0.956	0.418	0.459
MX2	0.927	0.949	0.441	0.509
HERC6	0.889	0.941	0.443	0.415
RSAD2	1.029	0.940	0.421	0.424
HERC5	1.064	0.937	0.415	0.436
SERPING1	0.898	0.918	0.461	0.565
PML	0.434	0.916	0.402	0.512
GBP1	0.984	0.904	0.538	0.576
CUL1	0.479	0.885	0.506	0.584
IFITM1	0.676	0.882	0.461	0.539
TYMP	0.531	0.877	0.462	0.646
CD2AP	0.412	0.875	0.545	0.516
APOL6	0.721	0.868	0.402	0.449
STAT1	0.801	0.865	0.526	0.497
SLFN5	0.997	0.862	0.498	0.535
FKBP15	0.401	0.858	0.491	0.491
GBP4	0.803	0.855	0.531	0.486
PLAC8	0.552	0.844	0.452	0.502
FAM8A1	0.427	0.842	0.572	0.526
ADAR	0.450	0.840	0.427	0.432
VRK2	0.463	0.832	0.521	0.496
IFI27	0.434	0.832	0.414	0.635
RP4-620F22.2	0.865	0.823	0.475	0.607
UBE2L6	0.423	0.823	0.401	0.493
RTCB	0.249	0.813	0.456	0.526
PARP11	0.423	0.812	0.570	0.463
KLHDC7B	0.621	0.810	0.531	0.686
SP140	0.814	0.806	0.468	0.407
PI4K2B	0.454	0.804	0.529	0.490
GBP5	0.888	0.800	0.489	0.586
RP11-609D21.3	0.925	0.969	-	0.410
DHX58	0.681	0.959	-	0.451
EPSTI1	0.860	0.956	-	0.476
IFI44L	0.899	0.948	-	0.410
CMPK2	0.885	0.945	-	0.430
AP001610.5	0.894	0.943	-	0.403
MX1	0.889	0.939	-	0.403
PARP12	0.649	0.935	-	0.473
PARP14	0.806	0.930	-	0.464
IRF7	0.722	0.928	-	0.532
SIGLEC1	0.929	0.928	-	0.425

Supplementary Table 5. List of the hub-genes identified in the brown signature (continued)

Gene	Fold change pSS vs HC	Module Membership	Gene Significance (sIgG)	Gene Significance (ESR)
ZBP1	0.923	0.917	-	0.529
NEXN	0.785	0.916	-	0.403
IFI35	0.517	0.910	-	0.499
EIF4B	-0.374	0.908	-	0.500
IFI6	0.792	0.904	-	0.451
ISG15	0.708	0.893	-	0.502
LY6E	0.613	0.888	-	0.504
DDX60L	0.667	0.884	-	0.421
ATP10A	0.716	0.874	-	0.440
EIF3L	-0.413	0.874	-	0.423
EIF2AK2	0.598	0.868	-	0.415
MOV10	0.515	0.867	-	0.420
HELZ2	0.609	0.866	-	0.469
CXCL10	0.858	0.860	-	0.459
SPATS2L	0.844	0.859	-	0.478
AGRN	0.674	0.851	-	0.471
ATP13A1	0.303	0.851	-	0.422
CXCR2P1	0.690	0.838	-	0.425
ISG20	0.770	0.836	-	0.634
QARS	-0.293	0.831	-	0.421
HSH2D	0.699	0.827	-	0.473
TCF4	0.514	0.826	-	0.490
FXR1	-0.236	0.820	-	0.463
OASL	0.639	0.816	-	0.515
AC009950.2	0.551	0.815	-	0.409
RP11-983P16.4	-0.314	0.802	-	0.476
IFIT3	1.106	0.950	0.404	-
PARP9	0.945	0.950	0.417	-
IFIT2	1.095	0.936	0.403	-
SAMD9L	1.015	0.926	0.432	-
SAMD9	0.866	0.895	0.466	-
BATF2	0.906	0.883	0.451	-
RP1-71H24.1	0.862	0.859	0.488	-
OAS1	0.883	0.847	0.471	-
TRIM5	0.613	0.831	0.404	-
SSB	0.314	0.822	0.404	-
DTX3L	0.796	0.816	0.449	-
PPM1K	0.574	0.814	0.415	-

Supplementary Table 6. List of the hub-genes identified in the yellow signature

Gene	Fold change pSS vs HC	Module Membership	Gene Significance (Schirmer)	Gene Significance (C4 levels)
FAM188A	0.410	0.936	0.462	0.488
DDOST	-0.213	0.921	0.582	0.407
KIAA0895L	0.510	0.908	0.441	0.438
TRIM38	0.255	0.906	0.423	0.492
RP11-6N17.10	0.313	0.856	0.424	0.438
MBOAT1	0.275	0.841	0.511	0.515
POLK	0.329	0.840	0.471	0.440
WDR55	0.262	0.906	-	0.463
RP11-488L18.4	0.409	0.894	-	0.412
GRIPAP1	0.348	0.878	-	0.472
IRF9	0.658	0.873	-	0.437
ZNF224	0.305	0.866	-	0.416
BLZF1	0.617	0.858	-	0.468
PRPF4B	0.256	0.853	-	0.408
APBB3	0.366	0.852	-	0.412
SP140L	0.275	0.852	-	0.554
DYM	-0.238	0.851	-	0.449
ATMIN	-0.166	0.849	-	0.478
NABP1	0.503	0.831	-	0.425
ATHL1	0.453	0.827	-	0.422
N4BP2L2	0.301	0.895	0.590	-
PPWD1	0.338	0.873	0.435	-
RSRP1	0.416	0.852	0.435	-
CAPRIN2	0.429	0.848	0.536	-
CLEC12B	0.641	0.844	0.538	-
TWF2	-0.191	0.832	0.449	-
RGL2	0.282	0.823	0.534	-
EBLN3	0.241	0.818	0.515	-
NOL8	0.275	0.817	0.432	-

Supplementary Table 7. List of the hub-genes identified in the turquoise signature

Gene	Fold change pSS vs HC	Module Membership	Gene Significance (C3 levels)
HERC4	0.463	0.929	0.546
STK38	0.477	0.929	0.506
GIMAP4	0.618	0.923	0.466
SEC22B	0.499	0.923	0.497
SLC2A1	-0.936	0.921	0.472
GIMAP8	0.568	0.921	0.562
DDX23	0.387	0.909	0.566
IRF2	0.520	0.909	0.447
STX7	0.619	0.909	0.552
SACM1L	0.441	0.908	0.583
SNX1	0.378	0.908	0.464
SEC23IP	0.415	0.907	0.466
METTL13	0.470	0.906	0.428
RNASEL	0.599	0.905	0.474
COPB2	0.455	0.902	0.516
NLRC4	0.610	0.901	0.530
VDAC2	-0.420	0.900	0.478
ZNF146	0.392	0.898	0.409
CHMP7	0.417	0.897	0.487
CLCN3	0.408	0.896	0.459
HMGCL	0.467	0.895	0.437
HOMEZ	0.635	0.895	0.411
DCAF12	0.496	0.895	0.553
PREPL	0.591	0.893	0.606
ARF1	-0.225	0.892	0.457
PCMTD1	0.383	0.891	0.619
MPHOSPH8	0.408	0.889	0.538
YIPF4	0.598	0.888	0.549
TRAFD1	0.423	0.883	0.467
RGS18	0.607	0.883	0.494
VTA1	0.364	0.882	0.409
MAPKAPK2	-0.380	0.877	0.646
PEX11B	0.448	0.875	0.561
NADK	0.392	0.875	0.420
GIMAP1	0.516	0.874	0.504
KLHL6	0.548	0.874	0.457
TRIP11	0.450	0.873	0.531
CCDC97	0.350	0.873	0.546
XPO1	0.311	0.873	0.509
SCIMP	0.480	0.871	0.401
ZC3H13	0.457	0.869	0.447
GLE1	0.305	0.868	0.460

Supplementary Table 7. List of the hub-genes identified in the turquoise signature (continued)

Gene	Fold change pSS vs HC	Module Membership	Gene Significance (C3 levels)
TMEM184C	0.437	0.868	0.416
IGSF6	0.404	0.868	0.415
SUPT20H	0.399	0.867	0.514
CARD8	0.394	0.867	0.607
WDR45B	-0.287	0.864	0.437
PARP4	0.476	0.864	0.514
BRPF1	-0.575	0.861	0.534
EDEM3	0.481	0.859	0.423
CBR4	0.480	0.859	0.527
USO1	0.287	0.859	0.521
PGBD2	0.601	0.859	0.489
ADNP2	-0.476	0.858	0.562
GIMAP7	0.458	0.857	0.448
NINJ1	-0.433	0.855	0.450
DNTTIP2	-0.493	0.854	0.417
BBS7	0.651	0.853	0.524
CNOT6	0.323	0.853	0.477
EMC2	0.498	0.853	0.484
XPC	0.381	0.848	0.444
CTTNBP2NL	0.443	0.848	0.556
ALYREF	-0.266	0.846	0.506
RP11-400F19.6	0.410	0.845	0.482
RNF168	-0.377	0.845	0.441
SLC25A33	-0.693	0.844	0.517
MNDA	0.505	0.844	0.447
TTI1	0.327	0.844	0.482
NCBP1	0.337	0.844	0.434
HMGA1	-0.450	0.844	0.525
TLR6	0.495	0.843	0.514
TTC33	0.570	0.842	0.494
RNF11	-0.310	0.839	0.479
RP11-361D15.2	-0.318	0.839	0.432
TRMT1L	0.506	0.838	0.469
IPO8	0.386	0.837	0.412
RBM14	0.410	0.837	0.472
NR1D1	-0.878	0.834	0.423
RBMXL1	-0.293	0.834	0.407
SLC35B3	0.463	0.832	0.571
TMEM60	0.564	0.830	0.408
MAPK14	0.303	0.827	0.425
PI4K2A	-0.679	0.827	0.526
PAFAH2	0.450	0.827	0.446

Supplementary Table 7. List of the hub-genes identified in the turquoise signature (continued)

Gene	Fold change pSS vs HC	Module Membership	Gene Significance (C3 levels)
P2RY13	0.497	0.826	0.539
GOLGA5	0.263	0.826	0.516
TTC9C	0.502	0.825	0.407
MAP2K3	-0.318	0.824	0.533
PLRG1	0.256	0.824	0.494
NFE2	0.593	0.822	0.413
RP11-332M2.1	-0.307	0.822	0.499
EPB41L4A-AS1	-0.507	0.821	0.419
VPS37B	-0.299	0.821	0.410
SLC30A5	0.310	0.820	0.410
PIGV	0.583	0.820	0.457
LMBRD2	0.556	0.820	0.517
SCAMP4	-0.276	0.818	0.473
FPR3	1.026	0.817	0.408
CARD8-AS1	0.500	0.816	0.477
MAPRE1	-0.231	0.815	0.446
C1orf112	0.635	0.813	0.488
TRAF3IP3	0.328	0.811	0.441
PQLC1	-0.285	0.810	0.487
TMEM189	-0.351	0.807	0.552
ID2	-0.574	0.804	0.511
BET1	0.533	0.804	0.419
SLC37A2	0.380	0.802	0.471

Supplementary Table 8. List of selected genes to represent the pSS-monocyte transcriptomic signature

Gene	Module	Module Membership	Fold change pSS vs HC	Gene significance
MX1	Brown	**0.9391**	**0.889**	sIgG (0.3608)<br>ESR (**0.4029**)
IFITM1	Brown	**0.8816**	**0.676**	sIgG (**0.4613**)<br>ESR (**0.5392**)
TNFSF10	Turquoise	**0.8855**	**0.717**	C3 (0.3959)
IRF2	Turquoise	**0.9086**	**0.520**	C3 (**0.4247**)
STX7	Turquoise	**0.9085**	**0.619**	C3 (**0.5521**)
TRIM38	Yellow	**0.9063**	0.255	C4 (**0.4916**)<br>Schirmer (**0.4227**)
IRF9	Yellow	**0.8731**	**0.658**	C4 (**0.4369**)<br>Schirmer (0.3401)
RPL5	Blue	**0.9483**	**-0.462**	-
RPL15	Blue	**0.9717**	**-0.421**	-
EEF1B2	Blue	**0.9362**	**-0.448**	-

Selection of the hallmark genes to represent each signature, and ultimately the transcriptomic profile of pSS monocytes, were based on the following criteria: 1) highest degree of correlation with each signature (high module membership); 2) highest fold-change of expression between pSS and HC; 3) gene significance > 0.4 in any of the clinical features. Parameters which meet the selection criteria are depicted in bold.
